# COVID-19 Vaccine for Children: Vaccination Willingness of Parents and Its Associated Factors—A Network Analysis

**DOI:** 10.3390/vaccines10071155

**Published:** 2022-07-20

**Authors:** Julia Barbara Krakowczyk, Alexander Bäuerle, Lars Pape, Theodor Kaup, Laura Nulle, Martin Teufel, Eva-Maria Skoda

**Affiliations:** 1 Clinic for Psychosomatic Medicine and Psychotherapy, LVR-University Hospital Essen, University of Duisburg-Essen, 45147 Essen, Germany; alexander.baeuerle@uni-due.de (A.B.); theodor.kaup@stud.uni-due.de (T.K.); laura.nulle@stud.uni-due.de (L.N.); martin.teufel@uni-due.de (M.T.); eva-maria.skoda@uni-due.de (E.-M.S.); 2 Center for Translational Neuro- and Behavioral Sciences (C-TNBS), University of Duisburg-Essen, 45147 Essen, Germany; 3 Department of Pediatrics II, University Hospital of Essen, University of Duisburg-Essen, 45147 Essen, Germany; lars.pape@uk-essen.de

**Keywords:** COVID-19, network analysis, vaccine acceptance, mental health

## Abstract

Different COVID-19 vaccines have been approved for underage children, so parents and caregivers currently face the decision of whether to vaccinate their children against COVID-19 or not. Due to the rather moderate vaccine acceptance among parents across different countries, the objective of the present study was to investigate the relationship between different psychological, demographic, and behavioral factors related to the acceptance of the COVID-19 vaccine for underage children among parents. In particular, vaccination attitudes, whether parents have been vaccinated against COVID-19 themselves, COVID-19 fear, attitude towards COVID-19 policy measures, governmental trust, subjective level of information, perceived risk of disease progression, and perceived risk of vaccine side effects were the variables of interest. The study adopted a cross-sectional study design, and the sample consisted of 2405 participants. A network analysis was conducted to investigate the associations and interconnection among these variables. The results showed that, in particular, compliance, confidence in the safety of vaccines, whether parents have been vaccinated against COVID-19 themselves, trust in the governmental system, fear of COVID-19, and the parents’ age were directly related to the acceptance of the COVID-19 vaccine for children. To increase compliance and confidence in the vaccines’ safety among parents, promotion campaigns should provide more information concerning the vaccines’ safety, particularly for younger parents who are not vaccinated against COVID-19 themselves.

## 1. Introduction

After the COVID-19 pandemic outbreak at the beginning of 2020, research and development efforts regarding effective prevention against the SARS-2 coronavirus soon resulted in the development of several vaccines by the end of 2020, which, shortly after, were approved and rolled out in many countries all over the world [1]. Vaccines play an important role in handling the COVID-19 pandemic, as they lower the risk of infection, severe disease progression, and even death [2]. Since the introduction of the first vaccines against COVID-19, there has been extensive research about their benefits and limitations [3,4,5]. The world has also faced several new emerging variants over the course of the pandemic that are assumed to reduce the vaccine’s impact concerning its transmissibility, pathogenicity, and COVID-19-related hospitalization rate [3,6]. The willingness to get vaccinated thus started to play a key role in governmental vaccination campaigns to decelerate the spread of the virus [7]. However, vaccination hesitancy, which, according to the World Health Organization (WHO), poses a threat to global health, started to become a considerable issue during the COVID-19 pandemic [8,9,10]. Medical considerations such as the vaccine’s novelty and medical preconditions seemed to have an effect on the willingness to get vaccinated [11]. In addition, other factors outside the range of medical-related concerns contributed to the likelihood of getting vaccinated, including educational degree, adherence to COVID-19-related measures, age, sex, the source of COVID-19-related information, and trust in authorities [12,13,14,15,16].

As new studies emerge evaluating the benefits and limitations of the vaccination of underage persons against COVID-19 [17,18,19], the discussion about vaccinating children is increasingly gaining the attention of parents and families. Vaccinating children against COVID-19 plays an important role in handling the COVID-19 pandemic. However, as these studies are still scarce, the question of whether to vaccinate children or not is a very sensitive topic [20]. Overall, research shows rather moderate acceptance of the COVID-19 vaccine for children across different countries [21,22]. Moderate vaccine acceptance has been observed in Germany as well, as only 54.1% of parents were willing to vaccinate their children against COVID-19 in 2021 [16]. According to the latest official measurement of age groups and their corresponding vaccination rates (accessed on 1 June 2022), only 66.8% of children between 12 and 17 have actually been vaccinated against COVID-19 [23]. Vaccine hesitancy seems to be even more prevalent among parents with younger children, as only 22.1% of children between 5 and 11 have been vaccinated against COVID-19 at least once [23]. Interestingly, a significant misinterpretation of vaccination risks and frequent belief in vaccination conspiracy theories were observed among parents in Germany [16]. In general, parents of underage children were observed to be less willing to be vaccinated themselves than childless parents, with this effect being more pronounced in mothers than in fathers [16]. Parents were also more likely to get vaccinated themselves than to vaccinate their children against COVID-19 [20,21,22,24,25,26,27].

Previous research in European countries shows that the decision of whether to vaccinate their children or not against COVID-19 is related to psychological, behavioral, and demographic factors. Demographic factors associated with vaccine acceptance include age, educational degree, gender, and working in the healthcare sector [27,28,29]. Age has been shown to be especially positively related to vaccine acceptance [27]. Psychological factors associated with vaccine acceptance among parents comprise the subjective level of information related to COVID-19, a positive attitude towards vaccines in general, a positive attitude towards policy measures, confidence in the safety of vaccines, a belief in collective responsibility, and fear of COVID-19 [2,27,28,29,30,31,32,33,34]. Considering behavioral factors, whether parents have been vaccinated against COVID-19 themselves or are willing to has been identified as a prominent predictor [35]. Psychological factors associated with vaccine hesitancy towards the COVID-19 vaccine for children included the perceived risk of possible side effects, concerns about vaccine safety, concerns about the fast development and qualification of the COVID-19 vaccine, and the contemporary lack of research in children [20,21,26,35,36].

Overall, research points towards a multifactorial picture with many interconnected variables related to the acceptance of the COVID-19 vaccine for children. However, the majority of previous research used isolated sets of selected variables to identify variables associated with vaccine acceptance. Hence, there is a lack of research investigating the interconnectedness of these variables together. Hence, it is of great importance to explore the relationship between those variables in a network. Network analysis offers a suitable approach to investigate this, as it assumes an interaction among distinct components within a system [37]. In the chosen approach, the variables in the network are called “nodes”, whereas the connections among them are referred to as “edges” [37]. Edge weights display the conditional dependencies between the retrospective nodes [38]. In general, network analysis is able to detect complex relationships and the interrelatedness between variables. Therefore, it is a suitable approach to investigate the interplay among demographic, behavioral, and psychological factors associated with vaccine acceptance among parents. Moreover, it is a promising approach to investigate COVID-19-related psychological variables, as a complex interaction among different variables is assumed during the COVID-19 pandemic [39].

The aim of the present study was to investigate the relationship and interconnectedness between different psychological, demographic, and behavioral factors related to the acceptance of the COVID-19 vaccine for underage children among parents. Specifically, the present study was interested in the relationship between vaccination attitudes, whether parents have been vaccinated against COVID-19 themselves, COVID-19 fear, attitude towards COVID-19 policy measures, governmental trust, subjective level of information related to COVID-19, perceived risk of disease progression if infected by COVID-19, and perceived risk of side effects of the COVID-19 vaccine. Although research exists that uses the network approach to assess variables associated with vaccination willingness on an individual level [33,39], to our knowledge, there is no respective research that investigates variables related to the acceptance of the COVID-19 vaccine particularly for children among parents. As new studies emerge that also recommend novel vaccines against COVID-19 for children and adolescents [40,41], parents and caregivers are facing the decision of whether to vaccinate their children or not. For that reason, the objective of the present study is to outline a comprehensive and interrelated image of various factors and attitudes that relate to this decision. The gained information helps to provide a better understanding for further research as well as for health authorities and professionals to respond to possible reservations in an adequate and purposive manner.

## 2. Materials and Methods

### 2.1. Study Design and Procedure

The study adopted a cross-sectional design. The Ethics Committee of the Faculty of Medicine of the University of Duisburg-Essen approved the setup of the study (20-9307-BO). An online survey using the software Unipark (Tivian XI GmbH) was distributed from 13 December 2021 to 31 January 2022 via social media, general practitioners, and pediatricians. Clinicians across different locations in Germany were contacted, informed about the purpose of the study, and invited to distribute the study among their patients. Participation in the survey was voluntary. The survey was administered in German. The inclusion criteria for participating were (1) age above 18, (2) sufficient knowledge of the German language, and (3) having a child under the age of 18. After obtaining electronic informed consent, participants filled in the survey, which took approximately 13.4 min to fill in. The completion rate was 72.45%. The data were processed anonymously, and withdrawal from the study was possible at any time. There was no form of reimbursement.

### 2.2. Materials

Attitudes toward vaccines were assessed with the German version of the 7c scale of vaccination readiness by Geiger et al., 2021 [42]. The 7c scale of vaccination readiness is an assessment tool that comprises 21 items assessing psychological antecedents of vaccination readiness. It is divided into seven subscales: confidence, constraints, complacency, calculation, collective responsibility, compliance, and conspiracy. The seven subscales comprise three items, which are based on a 7-point Likert scale (1 = strongly disagree; 7 = completely agree).

Confidence refers to the subjective trust in the safety of vaccines [43]. Constraints refer to personal or structural obstacles that make getting vaccinated difficult [43]. Complacency refers to the perceived risk of the corresponding disease [43]. Calculation describes the consideration of personal advantages and disadvantages when thinking of getting vaccinated [43]. Collective responsibility describes the subjective relevance of protecting others as an important factor when considering getting vaccinated [43]. Compliance describes the acceptance and adherence to vaccination policies [42]. Conspiracy describes the tendency to engage in conspiracy thinking [42].

The German version of the 7c scale is a validated instrument with adequate psychometric properties [42]. Previous research has shown that the seven psychological antecedents play a central role in vaccine acceptance, as they explain up to 85% of the variance in vaccination willingness [42]. The internal consistency was assessed in the current study as well. Overall, six of the seven subscales ranged between α = 0.82 and α = 0.92, which is considered a high to excellent internal consistency. However, the scale “calculated-reservation” did not show a high internal consistency (α = 0.45). Hence, the item “I only get vaccinated if there are no disadvantages for me” was deleted to achieve acceptable internal consistency (α = 0.6).

A positive attitude towards COVID-19 policy measures was assessed based on previous research [39,44,45,46], including the following items: “I feel like Germany is well prepared to handle the COVID-19 pandemic”, and “I think that all necessary measures are being taken to tackle COVID-19”. The items were based on a 7-point Likert scale (1 = strongly disagree; 7 = completely agree), and the internal consistency was acceptable to high (α = 0.78).

Based on previous research [39,44,45,46], the subjective level of information related to COVID-19 was assessed with the following items: “I feel well informed about COVID-19”, “I feel well informed about how to prevent an infection”, and “I understand the health authority’s advice regarding COVID-19”. The items were based on a 7-point Likert scale (1 = strongly disagree; 7 = completely agree), and the internal consistency was high (α = 0.81).

Based on previous research [39,44,45,46], governmental trust and COVID-19 fear were assessed with the following items: “COVID-19 displays a threat for me” and “I have trust in the governmental system”. The items were based on a 7-point Likert scale (1 = strongly disagree; 7 = completely agree).

The perceived risk of disease progression if the child becomes infected with COVID-19 was assessed with the following item: “How high do you estimate the risk that your child would suffer from a severe disease progress when being infected by COVID-19?”. The item was based on a visual analogue scale (VAS) from 0 to 100 (0 = not likely; 100 = very likely). The VAS was selected because it has shown good psychometric properties in online research [47].

The perceived risk of side effects of the COVID-19 vaccine was assessed with the following item: “How high do you estimate the risk that you/your child will experience side effects of the COVID-19 vaccine?”. The item was based on a visual analogue scale (VAS) from 0 to 100 (0 = not likely; 100 = very likely).

### 2.3. Data Analysis

R version 4.1.1 [48] was used to analyze the data. Missing values were excluded listwise. For a network analysis to achieve sufficient power, a minimum sample size between 250 and 350 for a network of around 20 nodes or fewer is required [49]. First, the network was estimated and visualized. As the data contained binary, ordinal, and continuous variables, a mixed graphical model was estimated [50]. Within a mixed graphical model, nodes represent the respective variables. The following variables were selected as nodes: age, governmental trust, COVID-19 fear, parental COVID-19 vaccine, confidence in the safety of vaccines, constraints to getting vaccinated, complacency, calculation, collective responsibility, compliance, conspiracy, subjective level of information related to COVID-19, attitude towards COVID-19 policy measures, acceptance of the COVID-19 vaccine for children, perceived risk of disease progression if infected by COVID-19, and perceived risk of side effects of the COVID-19 vaccine. In total, 16 nodes were included. In the network, edges represent the conditional dependencies among the retrospective variables using the Fruchtermann–Reingold algorithm [38]. The Least Absolute Shrinkage and Selection Operator (gLASSO) and the Extended Bayesian Information Criterion (EBIC) with a tuning parameter of 0.5 were applied [51,52].

Next, centrality indices were computed. Centrality indices evaluate the importance of a node to the network structure, as they determine influential nodes in the network. In the present study, the following centrality indices were estimated: degree centrality, strength, closeness, and betweenness. Degree centrality is the sum of all edges of a corresponding node. Node strength shows the sum of the edge weights of all edges of a corresponding node [53]. Node closeness assesses the average distance of a corresponding node to the other nodes [53]. Betweenness identifies the role of a corresponding node in connecting the other nodes [53].

Finally, different bootstrap procedures were used to assess the network’s stability and accuracy. An edge weight variation analysis was conducted to assess the accuracy of the network [54]. The interpretability of edge weight and node strength differences was assessed in the corresponding significance of edge weight and node strength analyses. To test the interpretability of the centrality indices, a correlation stability analysis was conducted.

## 3. Results

### 3.1. Sample Characteristics

The sample consisted of 2405 individuals. Among them, 93.3% identified as female, 6.3% identified as male, and 0.2% identified as non-binary. The age ranged between 20 and 67 years (M = 38.88; SD = 5.159). All individuals were parents of underage children. Most of them had one child (64.4%), 30.5% had two children, 4% had three children, and 0.9% had more than three children. Overall, 86.8% of the parents were vaccinated against COVID-19, whereas 13.2% were not. The majority of parents (71.4%) were willing to vaccinate their children against COVID-19, 9.3% were undetermined, and 19.3% were not willing. See Table 1 for detailed sample characteristics.

### 3.2. Network Estimation and Visualization

**Figure 1:**
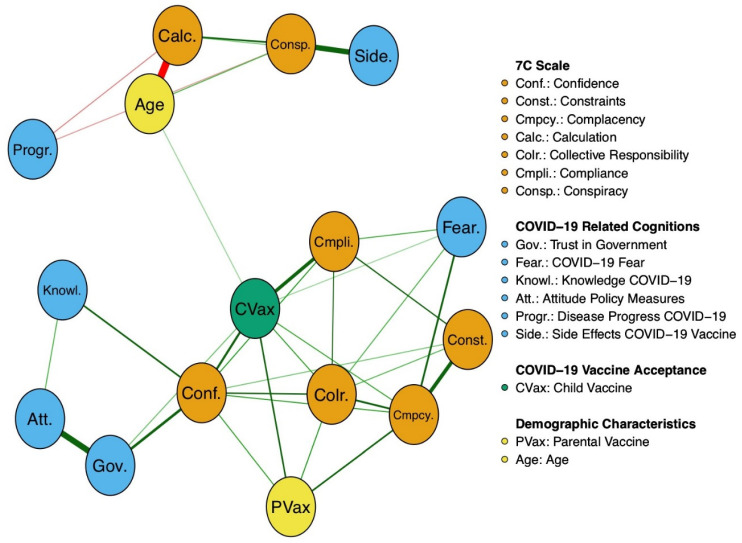
Visual MGM Network. The abbreviations within the network display the nodes. The connections between those nodes are referred to as edges. The thickness of the edges represents the edge weight, which is an indication of the strength of the edge. The thicker the edge, the higher the edge weight. Green edges represent positive associations, whereas red edges represent negative associations. The meanings of the variables’ abbreviations can be seen on the right side of the network display.

Out of 120 possible edges, 35 emerged (see Figure 1). Among them, 32 displayed positive associations, whereas 3 displayed negative ones. A graphical representation of the network with displayed edge weights can be found in the Supplementary Material (Figure S1). The strongest edge weights were found between age and calculation (−0.55), governmental trust and attitude towards COVID-19 policy measures (0.46), conspiracy and perceived risk of severe side effects of the COVID-19 vaccine (0.42), complacency and constraints to getting vaccinated (0.32), acceptance of the COVID-19 vaccine for children and compliance (0.33), governmental trust and confidence in the safety of vaccines (0.25), and confidence in the safety of vaccines and acceptance of the COVID-19 vaccine for children (0.22).

The strongest edges associated with acceptance of the COVID-19 vaccine for children were compliance (0.33), confidence in the safety of vaccines (0.22), parental COVID-19 vaccine (0.18), collective responsibility (0.14), and complacency (0.13). Governmental trust (0.08), COVID-19 fear (0.06), and age (0.06) shared weaker but also significant edges with the acceptance of the COVID-19 vaccine for children.

Attitude towards COVID-19 policy measures, the subjective level of information related to COVID-19, constraints to getting vaccinated, calculated reservation, conspiracy, fear of side effects of the COVID-19 vaccine, and fear of severe disease progression did not share any significant edges with acceptance of the COVID-19 vaccine for children.

### 3.3. Centrality Indices

**Figure 2:**
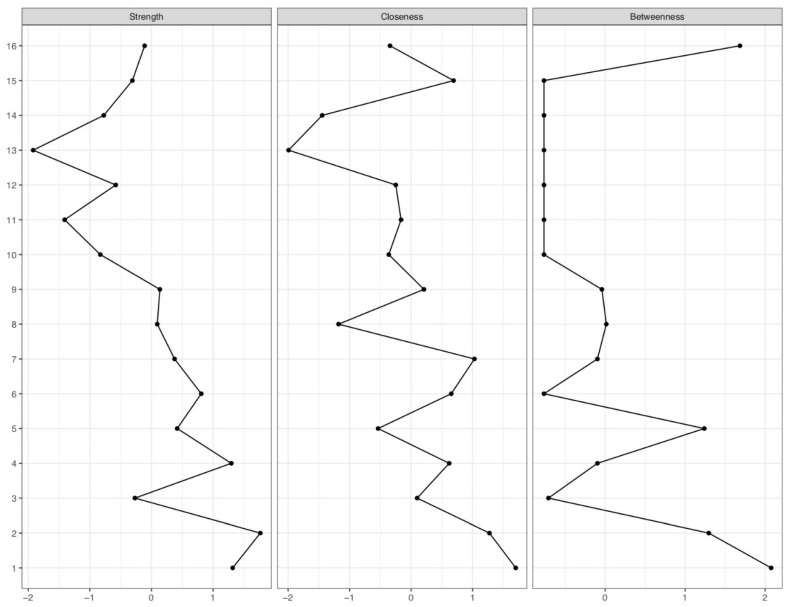
Display of centrality indices strength, closeness, and betweenness. The numbers on the y-axis represent the allocated nodes numbered sequentially. The x-axis represents z-values associated with the centrality indices. The higher the z-value, the higher the centrality index of the respective variable. For a node legend, see Figure 1. For a detailed explanation of the centrality indices, see Section 2.3 Data Analysis.

The nodes with the highest degree centrality were confidence in the safety of vaccines, collective responsibility, and acceptance of the COVID-19 vaccine for children. The highest strength index was for acceptance of the COVID-19 vaccine for children, parental COVID-19 vaccine, compliance, and complacency (see Figure 2). The highest closeness index was for parental COVID-19 vaccine, acceptance of the COVID-19 vaccine for children, complacency, and confidence in the safety of vaccines (see Figure 2). The highest betweenness index was for acceptance of the COVID-19 vaccine for children, age, and calculated reservation (see Figure 2).

### 3.4. Bootstrapping Procedures

Bootstrapping procedures were conducted for edge weight variation, the significance of edge weight and node strength differences, and the correlation stability of the centrality indices. The bootstrapping procedures showed high stability and interpretability of both the network and centrality indices. Additionally, the accuracy of the edge-weight parameters was validated. A detailed description of the bootstrap procedures can be found in the Supplementary Material (Figures S2–S5).

## 4. Discussion

The aim of the present study was to investigate the relationship and interconnectedness between different psychological, demographic, and behavioral factors related to the acceptance of the COVID-19 vaccine for children among parents using network analysis. Specifically, vaccination attitudes, whether parents have been vaccinated against COVID-19 themselves, COVID-19 fear, attitude towards COVID-19 policy measures, governmental trust, subjective level of information related to COVID-19, perceived risk of severe disease progression if infected by COVID-19, and perceived risk of side effects of the COVID-19 vaccine were the variables of interest. Overall, an accurate and well-interpretable network emerged. The variables whether parents have been vaccinated against COVID-19 themselves, acceptance of the COVID-19 vaccine for children, COVID-19 fear, confidence in the safety of vaccines, constraints to getting vaccinated, complacency, collective responsibility, and compliance showed high interconnectedness among each other. The variables calculated reservation, conspiracy, perceived risk of side effects of the COVID-19 vaccine, and fear of severe disease progression if infected by COVID-19 formed sparse connections and were rather connected to each other. Moreover, they were not related to the acceptance of the COVID-19 vaccine for children.

It was shown that acceptance of the COVID-19 vaccine for children was most strongly associated with compliance. Based on Geiger et al. [42], compliance describes someone’s overall acceptance and adherence to vaccination policies. This is in line with previous research showing a positive association between parents’ compliance with pandemic-related policies and the decision to vaccinate their children against COVID-19 [24,55]. Moreover, previous research found that this association was present for other vaccines in addition to the COVID-19 vaccine [56]. Hence, the tendency to accept the COVID-19 vaccine for children seems to be related to the overall adherence to vaccination policies [57].

Another psychological factor that was associated with both compliance and acceptance of the COVID-19 vaccine for children was the internal belief in the safety of the respective vaccine [58]. In the present study, confidence in the safety of vaccines was, next to compliance, most strongly associated with the acceptance of the COVID-19 vaccine for children. This finding underlines the interconnection between these variables, which has also been shown in past research [24,59,60,61]. Overall, these findings suggest that a belief in the safety of vaccines, compliance with vaccination policies, and acceptance of the COVID-19 vaccine for children display a network of interdependent variables.

It was shown that whether parents were vaccinated against COVID-19 themselves was related to the acceptance of the COVID-19 vaccine for children. This is in line with past research, as similar findings have been found in different countries as well [35,62,63]. It is suggested that parents’ decision of whether to vaccinate their children or not might be partially driven by the same motivations, such as the protection of others, that equally shape their individual vaccination decision. This assumption is highlighted by the results of the present study that collective responsibility was equally related to whether parents have been vaccinated against COVID-19 themselves and the acceptance of the COVID-19 vaccine for children.

Collective responsibility was identified as a central variable in the network of the present study. Based on the centrality indices, it was shown that, among all variables, collective responsibility displayed the most connections to all other variables. Similar findings have been found in past research as well [63,64,65]. Overall, these findings illustrate the determining role of an internal feeling of collective responsibility in vaccine acceptance. This suggests that the subjective relevance of protecting others plays an important role in vaccine acceptance. Interestingly, collective responsibility was strongly related not only to the acceptance of the COVID-19 vaccine but also to compliance. This indicates that, among parents, the subjective responsibility towards others that comes with the vaccination of their children is associated with adherence to vaccination policies. This might shed light on why parents are willing to vaccinate their children against COVID-19 even though research about the effectiveness and long-term risks is still scarce [66,67].

Another relationship emerged between acceptance of the COVID-19 vaccine for children and trust in the governmental system. This is in line with past research that also confirmed a relationship between vaccine acceptance and trust in the government [68]. This relationship has been found not only in regard to specific COVID-19 vaccine acceptance but also for overall vaccine acceptance [30]. This relationship displays the importance of the government during the COVID-19 pandemic and its associated vaccination policy. It is suggested that the government’s handling of the COVID-19 pandemic and the resulting trust in the government might potentially influence vaccine acceptance. Hence, it is essential that the government maintains a consistent and evidence-based approach to the vaccination policy associated with the COVID-19 pandemic.

In the present study, COVID-19 fear was associated with the acceptance of the COVID-19 vaccine for children. This is in line with previous research, and it indicates that parents’ fear of COVID-19 seems to be a motivator for vaccinating their children against the disease [24,69,70]. Interestingly, this relationship was only found for acceptance of the vaccine for children and not for whether parents have been vaccinated against COVID-19 themselves. Past research that also used network analysis did not find a significant relationship between personal vaccination willingness and COVID-19-related fear either [39]. This suggests that individual vaccination considerations are not directly associated with COVID-19-related fear, whereas this is the case for vaccination considerations related to children.

In addition to the previously discussed psychological and behavioral factors related to vaccine acceptance among parents, the present study also investigated the role of demographic factors, particularly age. Past research has already displayed a relationship between parents’ age and vaccination acceptance for their children, as a higher parental age positively predicted child vaccination against COVID-19 [35,60,71,72]. In the present study, age was also positively associated with vaccine acceptance, showing that older parents were more likely to vaccinate their children against COVID-19. However, compared to the above-mentioned psychological and behavioral variables, this relationship was rather weak. This suggests that although age is associated with vaccine acceptance, it might not be the most determining variable related to vaccination willingness. On closer analysis, the only variable that age was additionally associated with was calculation. Calculation refers to the consideration of personal advantages and disadvantages related to the vaccination decision [42]. This indicates that older parents showed less consideration of personal consequences than younger parents while showing a higher willingness to vaccinate their children. This is in line with past research showing that older age groups reported fewer vaccine-specific concerns, less need for more information, and fewer anti-vaccine attitudes [73].

Interestingly, the present study did not find a significant relationship between acceptance of the COVID-19 vaccine for children and a belief in conspiracy theories. This is not in line with past research, which identified a belief in conspiracy theories as a predictor for vaccination hesitancy during the COVID-19 pandemic [74]. In addition, past research from Germany showed that a belief in conspiracy theories was prevalent among parents and negatively related to vaccination willingness [16]. However, a significant association between those variables could not be found in the present study. This might be because a belief in conspiracy theories might not be such a prominent variable, compared to the previously discussed variables, related to vaccine acceptance. Another explanation might be that a tendency to engage in conspiracy thinking was not as strongly represented in the sample of the present study. Hence, further research is needed to investigate the role of the internal belief in conspiracy theories related to vaccine acceptance and to examine possible predictors.

The present study is the first study to investigate different variables that are associated with the acceptance of the COVID-19 vaccine for children among parents using network analysis. The wide range of psychological, behavioral, and demographic variables and the visualized display of interconnections among them offer a valuable contribution to better understanding the complex network of factors associated with vaccination willingness among parents. However, some limitations need to be taken into account. First, this study derived its data from online channels and professionals. Hence, a possible selection bias needs to be considered due to the convenience sample. Similarly, the uneven distribution of female participants and male participants also needs to be taken into account, as 93.3% identified as female. The majority of parents had also completed a college degree. Moreover, the current study did not assess the national background or ethnicity of the participants, so there is a risk of a possible cultural bias. Hence, further research using different samples and research in other countries is needed to detect possible parallels or disparities. The chosen cross-sectional study design and the network analysis approach were applied for exploratory purposes and thus could not detect causalities. For the sake of better understanding, further studies that investigate causal relationships are encouraged.

Overall, the present study offers a better comprehension of the interrelatedness among different variables associated with acceptance of the COVID-19 vaccine for children among parents. As parents and caregivers currently face the decision of whether to vaccinate their children or not, the present study outlines a comprehensive and interrelated image of various factors and attitudes that relate to this decision. The gained information helps to provide a better understanding for further research as well as for health authorities and professionals to respond to possible reservations in an adequate and purposive manner. Based on the findings of the present study, policy vaccination campaigns should provide adequate and evidence-based information concerning the COVID-19 vaccine for children, particularly for younger parents.

## 5. Conclusions

The present study investigated the relations between various psychological, behavioral, and demographic variables that are associated with the acceptance of the COVID-19 vaccine for children among parents. The network analysis approach was applied to inspect the complex interconnections between them. It was shown that, in particular, compliance, confidence in the safety of vaccines, whether parents have been vaccinated against COVID-19 themselves, trust in the governmental system, fear of COVID-19, and the parents’ age were directly related to the acceptance of the COVID-19 vaccine for children. Other factors such as personal constraints, attitude towards COVID-19 policy measures, and the subjective level of information related to COVID-19 were not directly related to acceptance of the COVID-19 vaccine for children but indirectly connected to it through variables such as confidence in the safety of vaccines and the internal belief of collective responsibility. The variables calculated reservation, belief in conspiracy theories, perceived risk of vaccine side effects, and perceived risk of disease progression if infected by COVID-19 were not significantly related to vaccine acceptance. Overall, the present study offers a contribution to a broader overview and a better comprehension of possible factors that are related to acceptance of the COVID-19 vaccine for children and how these variables are interrelated. To increase compliance and confidence in vaccine safety among parents, promotion campaigns should provide more information concerning vaccine safety, particularly for younger parents who are not vaccinated against COVID-19 themselves. In the context of rolling out new vaccines in the future, the results point to the importance of evidence-based vaccine promotion campaigns to support compliance, confidence in the safety of vaccines, overall vaccine acceptance, and trust in the governmental system among the population.

## Figures and Tables

**Table 1:** Demographic Characteristics of the Sample (N = 2405).

		N	%
Gender			
	Female	2252	93.6
	Male	148	6.2
	Diverse	5	0.2
Level of education			
	University degree	1371	57
	High school degree	315	13.1
	Higher middle school degree	105	4.4
	Lower middle school degree	597	24.8
	Other form of schooling	4	0.1
Residence area			
	Urban area (population size > 20,000)	853	35.5
	Rural area (population size < 20,000)	1552	64.5
Marital status			
	Single	73	3.0
	Married	2009	83.5
	In a relationship	225	9.4
	Separated/divorced	93	3.9
	Widowed	3	0.1
	Other	2	0.1
COVID-19 vaccine			
	Yes	2088	86.8
	No	317	13.2
Health status			
	Physical illness	486	20.2
	Mental illness	212	8.8
Number of children < 18			
	1	1553	64.4
	2	734	30.5
	3	97	4.0
	More than 3	21	0.9
Age of children			
	5	766	31.9
	6	539	22.4
	7	480	20.0
	8	414	17.2
	9	359	14.9
	10	302	12.6
	11	306	12.7
	>12	508	21.1

## Data Availability

Data are available on request due to privacy restrictions.

## Supplementary Materials

The following supporting information can be downloaded at: https://www.mdpi.com/article/10.3390/vaccines10071155/s1. Figure S1: Visualized MGM Network With Displayed Edge Weights; Figure S2: Correlation Stability Analysis of the Centrality Indices; Figure S3: Analysis of Edge Weight Variation; Figure S4: Significance of Edge Weight Differences; Figure S5: Significance of Node Strength Differences; Table S1: Distribution of Participants by Region (Germany, N = 2405).
