# Supplementary material for: COVID-19 Vaccine for Children: Vaccination Willingness of Parents and Its Associated Factors—A Network Analysis
Source: Vaccines (Basel). 2022 Jul 20;10(7):1155. doi: 10.3390/vaccines10071155 (PMC9320709; doi:10.3390/vaccines10071155)
Supplement: Supplementary file 1 [file vaccines-10-01155-s001.zip › vaccines-1776794-supplementary.pdf]

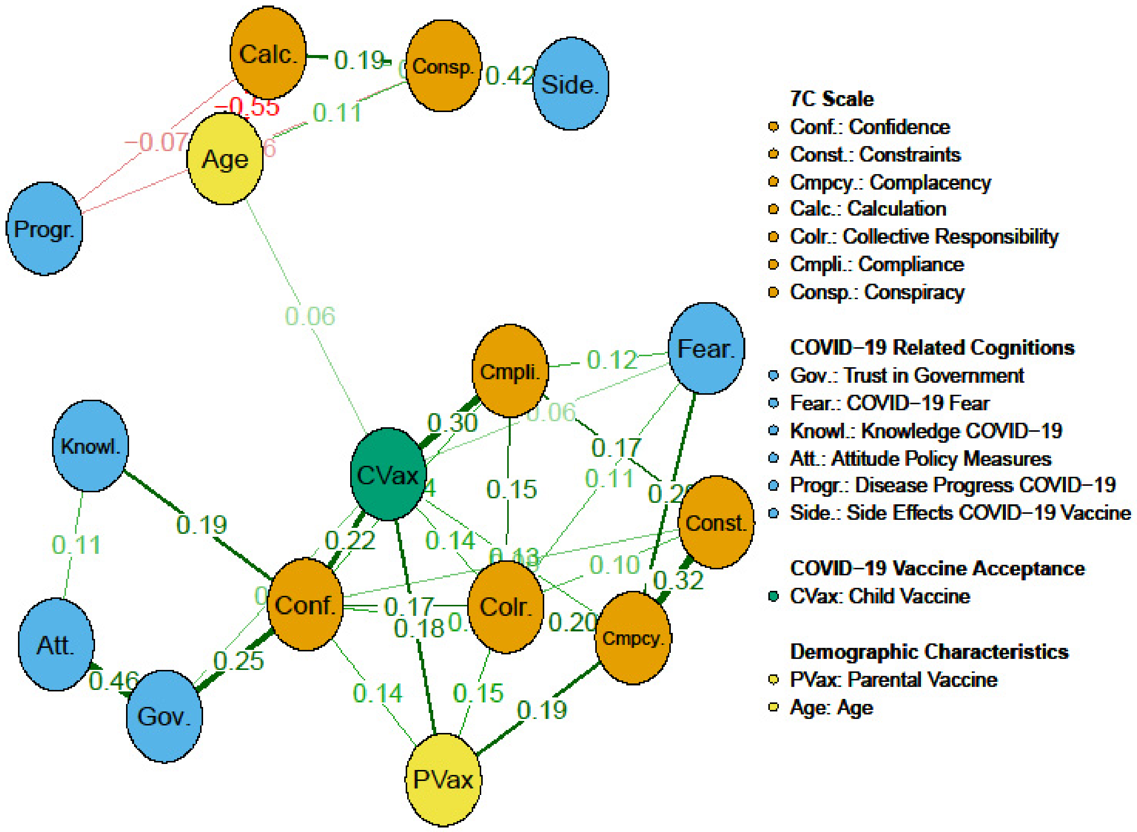

Figure S1

Visualized MGM Network With Displayed Edge Weights

The abbreviations within the network display the nodes. The connections between those nodes are referred to as edges. The thickness of the edges represents the edge weight which is an indication of the strength of the edge. The thicker the edge, the higher the edge weight. Green edges represent positive associations, whereas red edges represent negative associations. The meaning of the variables' abbrevations can be seen on the right side of the network display.

Figure S2

Correlation Stability Analysis of the Centrality Indices

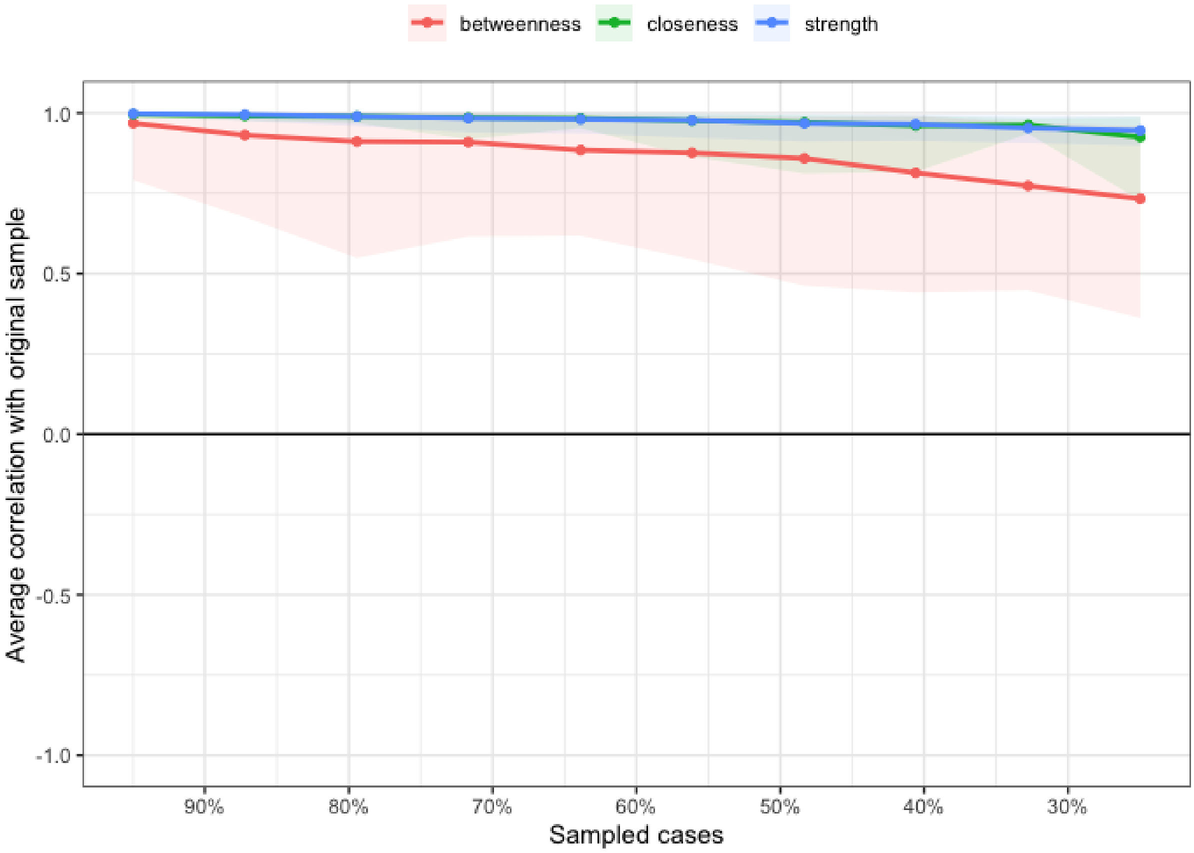

Figure S3

Analysis of Edge Weight Variation

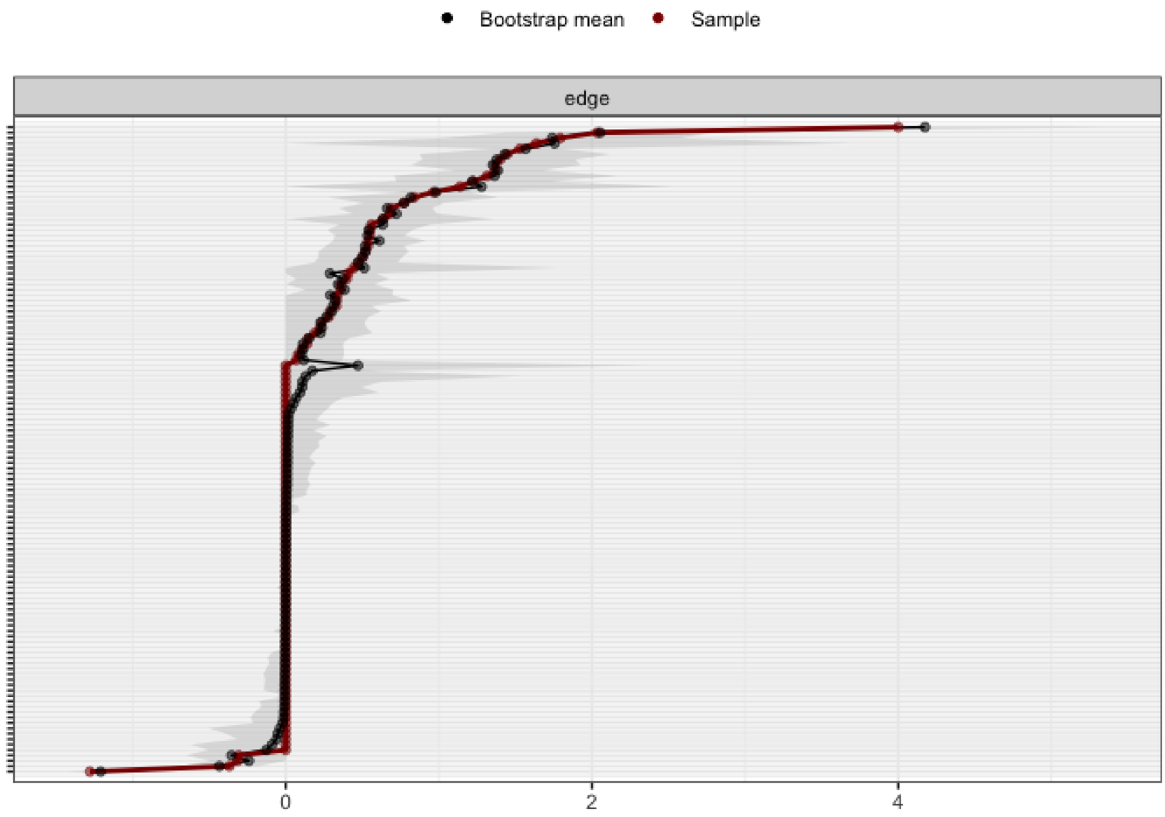

Figure S4

Significance of Edge Weight Differences

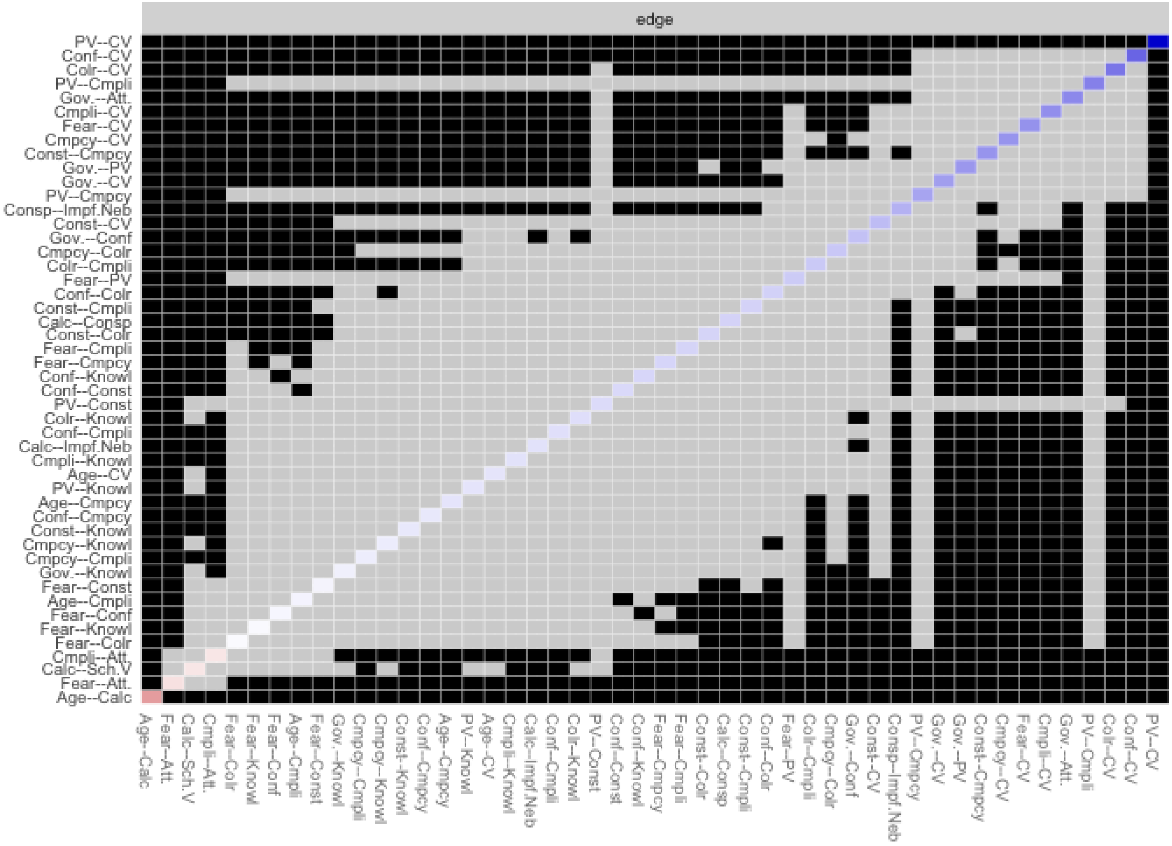

Figure S5

Significance of Node Strength Differences

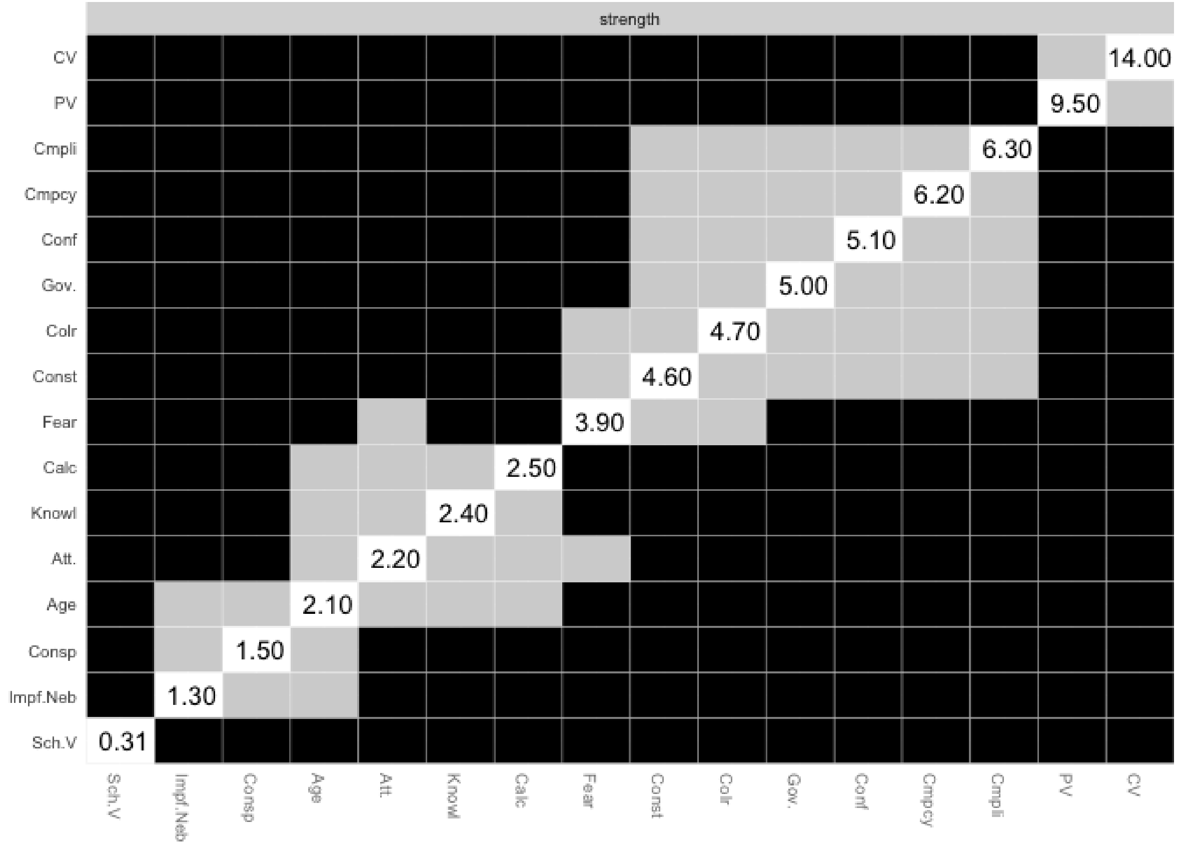

Table S1

Distribution of Participants by Region (Germany, N = 2405)

Bundesland	N	%
Baden-Württemberg	325	13.5
Bayern	347	14.4
Berlin	101	4.2
Brandenburg	38	1.6
Bremen	11	0.5
Hamburg	71	3.0
Hessen	159	6.6
Mecklenburg-Vorpommern	16	0.7
Niedersachsen	263	10.9
Nordrhein-Westfalen	716	29.8
Rheinland-Pfalz	106	4.4
Saarland	22	0.9
Sachsen	66	2.7
Sachsen-Anhalt	28	1.2
Schleswig-Holstein	113	4.7
Thüringen	23	1.0
